# Rapid identification of early infections in febrile patients after CD19 target CAR-T cell therapy for B-cell malignancies

**DOI:** 10.1186/s12967-024-05308-2

**Published:** 2024-07-02

**Authors:** Lian-Fang Pu, Hui-Min Zheng, Xiang-Jiang Feng, Alice Charwudzi, Xue Liang, Lin-Hui Hu, Yang-Yang Ding, Ze-Lin Liu, Ya Liao, Shu-Dao Xiong

**Affiliations:** 1 Hematological Lab, The Second Affiliated Hospital of Anhui Medical University, Hefei, Anhui People’s Republic of China; 2 Department of Hematology, The Second Affiliated Hospital of Anhui Medical University, Hefei, Anhui People’s Republic of China; 3 University of Cape Coast School of Medical Sciences, Cape Coast, Ghana; 4 Research Center for Translational Medicine, The Second Hospital of Anhui Medical University, Hefei, Anhui People’s Republic of China

**Keywords:** Refractory/relapsed B-cell malignancies, Chimeric antigen receptor T cell therapy, Early infection, Cytokine release syndrome, Inflammatory biomarkers

## Abstract

**Background:**

CD19-targeted chimeric antigen receptor T (CAR-T) cell therapy stands out as a revolutionary intervention, exhibiting remarkable remission rates in patients with refractory/relapsed (R/R) B-cell malignancies. However, the potential side effects of therapy, particularly cytokine release syndrome (CRS) and infections, pose significant challenges due to their overlapping clinical features. Promptly distinguishing between CRS and infection post CD19 target CAR-T cell infusion (CTI) remains a clinical dilemma. Our study aimed to analyze the incidence of infections and identify key indicators for early infection detection in febrile patients within 30 days post-CTI for B-cell malignancies.

**Methods:**

In this retrospective cohort study, a cohort of 104 consecutive patients with R/R B-cell malignancies who underwent CAR-T therapy was reviewed. Clinical data including age, gender, CRS, ICANS, treatment history, infection incidence, and treatment responses were collected. Serum biomarkers procalcitonin (PCT), interleukin-6 (IL-6), and C-reactive protein (CRP) levels were analyzed using chemiluminescent assays. Statistical analyses employed Pearson’s Chi-square test, t-test, Mann–Whitney U-test, Kaplan–Meier survival analysis, Cox proportional hazards regression model, Spearman rank correlation, and receiver operating characteristic (ROC) curve analysis to evaluate diagnostic accuracy and develop predictive models through multivariate logistic regression.

**Results:**

In this study, 38 patients (36.5%) experienced infections (30 bacterial, 5 fungal, and 3 viral) within the first 30 days of CAR T-cell infusion. In general, bacterial, fungal, and viral infections were detected at a median of 7, 8, and 9 days, respectively, after CAR T-cell infusion. Prior allogeneic hematopoietic cell transplantation (HCT) was an independent risk factor for infection (Hazard Ratio [HR]: 4.432 [1.262–15.565], P = 0.020). Furthermore, CRS was an independent risk factor for both infection ((HR: 2.903 [1.577–5.345], P < 0.001) and severe infection (9.040 [2.256–36.232], P < 0.001). Serum PCT, IL-6, and CRP were valuable in early infection prediction post-CAR-T therapy, particularly PCT with the highest area under the ROC curve (AUC) of 0.897. A diagnostic model incorporating PCT and CRP demonstrated an AUC of 0.903 with sensitivity and specificity above 83%. For severe infections, a model including CRS severity and PCT showed an exceptional AUC of 0.991 with perfect sensitivity and high specificity. Based on the aforementioned analysis, we proposed a workflow for the rapid identification of early infection during CAR-T cell therapy.

**Conclusions:**

CRS and prior allogeneic HCT are independent infection risk factors post-CTI in febrile B-cell malignancy patients. Our identification of novel models using PCT and CRP for predicting infection, and PCT and CRS for predicting severe infection, offers potential to guide therapeutic decisions and enhance the efficacy of CAR-T cell therapy in the future.

**Supplementary Information:**

The online version contains supplementary material available at 10.1186/s12967-024-05308-2.

## Introduction

Chimeric antigen receptor-modified T (CAR-T) cell therapy has emerged as a groundbreaking approach in cellular immunotherapy, demonstrating remarkable efficacy against refractory/relapsed (R/R) B-cell malignancies [1–4]. Despite its promising potential, the widespread adoption of CAR T-cell therapy faces significant obstacles due to the occurrence of severe and, at times, life-threatening toxicities [5, 6]. These toxicities encompass cytokine release syndrome (CRS), immune effector cell-associated neurotoxicity syndrome (ICANS), and early immune effector cell-associated hematotoxicity (ICAHT) [7–9]. Additionally, the delayed onset of cytopenias and long-term consequences such as B-cell aplasia and antibody deficiencies elevate the risk of infections, potentially leading to fatal outcomes post CAR-T cell therapy [10, 11]. Notably, severe infections are linked to early mortality in CAR-T therapy, underscoring the critical importance of promptly identifying early infections in febrile patients following CAR-T cell therapy for B-cell malignancies.

Biomarkers for early infection diagnosis, including procalcitonin (PCT), Interleukin-6 (IL-6), and (CRP), have been extensively studied [12–14]. While these markers show promise, they also have limitations. CRP lacks optimal sensitivity and specificity, IL-6 lacks specificity for hematological malignancies despite its rapid post-infection rise, and PCT may be influenced by coexisting conditions like tumors and thyroid diseases [15, 16]. but, combining these biomarkers may improve infection diagnosis accuracy [17, 18].

Clinically, differentiating between infection and CRS poses a challenge due to shared symptoms such as fever and elevated inflammatory factors [19]. Earlier investigations have indicated that 23–42% of patients undergoing CAR T-cell therapy experienced infections, primarily of bacterial origin (17–32%), within the initial month post-treatment initiation, coinciding with the onset of CRS [11]. Given the distinct therapeutic approaches for CRS and infections, an early differentiation between the two becomes imperative during CAR T-cell therapy. However, investigation of infection diagnosis in CAR-T therapy patients by the utility of inflammatory markers was limited [20, 21], and an urgent need to establish rapid and effective protocols for the early identification of infections, especially during CAR-T cell therapy.

The objective of our study was to understand the characteristics and identify crucial indicators for the early detection of infections in febrile patients within 30 days post CAR-T cell infusion (CTI) for B-cell malignancies. We conducted a retrospective analysis of infections within the initial 30 days post CD19 CAR-T cell immunotherapy in 104 patients with R/R B-cell malignancies. Subsequently, we assessed the levels of serum procalcitonin (PCT), interleukin-6 (IL-6), and C-reactive protein (CRP) at the onset of fever after CTI. Finally, we evaluated the value of these indicators for the prompt diagnosis of infections after CTI. Through our study, we aim to mitigate the deleterious effects of infections and optimize the efficacy of CAR-T cell therapy.

## Materials and methods

### Patient population

**Fig. 1:**
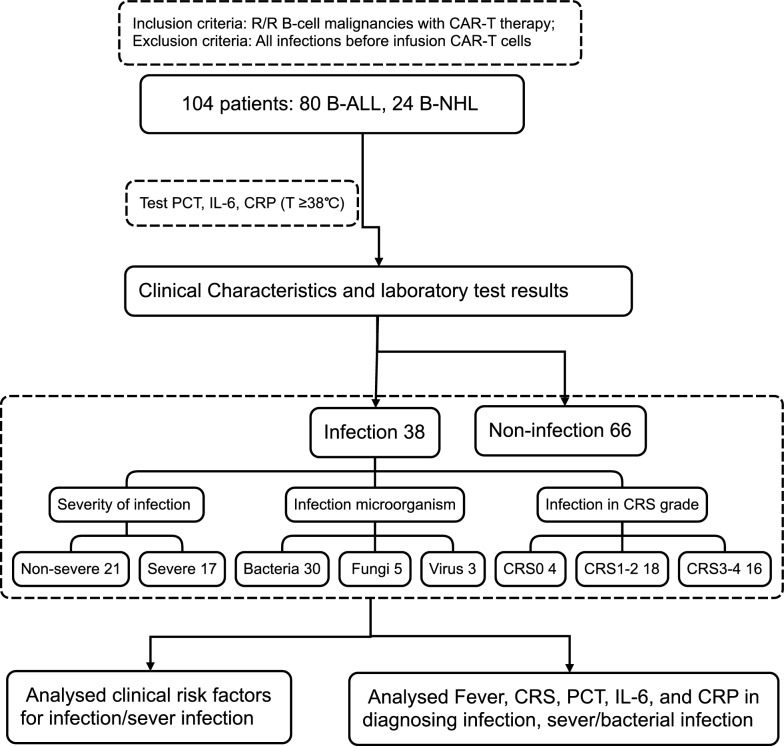
The flowchart of the study design. *B-ALL* B-cell acute lymphoblastic leukemia, *B-NHL* B-cell non-hodgkin lymphoma, *CRS* cytokine release syndrome, *PCT* procalcitonin, *IL-6* interleukin-6, *CRP* C-reactive protein

We conducted a retrospective cohort study involving 104 consecutive patients diagnosed with R/R B-cell malignancies who underwent CAR-T cell therapy at the Hematology Department of the Second Affiliated Hospital, Anhui Medical University, China, during the period from November 2015 to June 2023. The criteria for selecting patients with R/R B-cell malignancies have been previously outlined, and a detailed description of the therapy procedures can be found elsewhere [22]. The study design flowchart is depicted in Fig. 1. The study received approval from the Institutional Review Board of the Second Affiliated Hospital of Anhui Medical University. Prior to their inclusion in the study, all participants provided written informed consent, adhering to the principles of the Declaration of Helsinki.

### Clinical data collection

Clinical data, encompassing parameters such as age, gender, CRS, ICANS, treatment history, occurrences of infections, and therapeutic responses, were meticulously extracted from medical records. The grading and diagnosis of CRS adhered to the consensus grading system established by the American Society for Blood and Marrow Transplantation (ASBMT) [23]. The onset of CRS, defined by the manifestation of symptoms, including fever ≥ 38.0 °C, marked the first day of assessment. All infections before infusion were excluded. For the evaluation of infections, we employed a 5-point scale aligned with the Common Terminology Criteria of Adverse Events (CTCAE, version 4.0.3) was employed [21, 22]. Infection severity was classified as mild, moderate, severe, life-threatening, and fatal, as reported previously [11]. Mild and moderate infections, requiring no treatment or only oral treatment, were categorized as non-severe infections. Severe infections necessitated intravenous antimicrobial therapy or were associated with clinical circumstances considered severe. Life-threatening infections were characterized by symptoms deemed life-threatening, while fatal infections significantly contributed to death [11, 21]. Cases resulting in severe infections, life-threatening infections, and fatal infections were collectively categorized as severe infections. The incidence of infection events during the initial 30 days post-CAR-T cell therapy was determined based on relevant laboratory findings (microbiological, hematological, and histopathological) and radiographic results, thoroughly scrutinized to establish a diagnosis [24, 25]. The day of diagnostic testing was considered as the time of occurrence for the respective infection events.

### Serum indicators analyses

Serum indicators analysis entailed aseptic blood sample collection from all patients within 30 min of the onset of fever (defined as a body temperature ≥ 38 ℃). The levels of serum PCT and IL-6 were determined using the appropriate immunoassay employing a sandwich technique (Elecsys BRAHMS PCT assay, Elecsys IL-6 assay, Roche, Switzerland) and a chemiluminescent detection system (cobas e 601, Roche, Switzerland) in accordance with the instructions provided by the manufacturer. Additionally, the concentration of CRP in the serum was assessed using the IMMAGE 800 specific protein analysis system (Beckman Coulter Company, USA) following the manufacturer's protocol.

### Statistical analysis

We presented summary statistics for categorical variables and medians with interquartile ranges (IQR) for continuous variables, stratified based on the underlying disease type. Group comparisons were conducted using Pearson’s χ^2^-test for categorical data and either the unpaired sample t-test or Mann–Whitney U-test for numerical data, based on their distribution. Time-to-event analysis for infection was illustrated through cumulative incidence curves, utilizing Kaplan–Meier estimates, with observation time censored at the last follow-up date or date of death.

Clinical risk factors for infection were assessed using Cox proportional hazards regression analysis. The clinical variables examined included gender, age, diagnosis, extramedullary disease (EMD), more than 10 prior chemotherapy sessions, more than 2 relapses or cases of refractory disease, previous allogeneic hematopoietic stem cell transplantation (HCT), CRS grade, ICANS, and tocilizumab or corticosteroids for CRS treatment. Initially, various clinical characteristics were analyzed individually using univariate analysis. We then applied multivariable Cox proportional hazards regression using the enter method to determine whether any of these clinical characteristics were significant risk factors for infection. Correlation analysis of the indicators for fever, PCT, IL-6, CRP and CRS was conducted. The choice between Pearson's or Spearman's correlation was made based on the distribution of the data. Diagnostic accuracy was evaluated by computing the areas under the Receiver Operating Characteristic (ROC) curves (AUC), and optimal cut-off values were determined using the Youden's index (J = max [sens + spec − 1]).

Univariate logistic regression analyses were conducted, followed by stepwise multivariate logistic regression analyses using an entry criterion of P < 0.1. Based on the results of the multivariate analyses, we established a diagnostic mathematical model. Using this model, we performed ROC curve analysis and calculated the predictive value to assess the effectiveness of the biomarker combination. All statistical analyses were two-sided, with a significance level set at P < 0.05. Statistical software used for the analysis included GraphPad Prism 8 (GraphPad Software, Inc.) and IBM SPSS Statistics Version 23.0 (IBM Corporation, Armonk, NY, USA).

## Results

### Characteristics of patients

In our study, we enrolled 104 patients diagnosed with R/R B-cell malignancies, comprising 80 patients (76.9%) with B-cell acute lymphoblastic leukemia (B-ALL) and 24 patients (23.1%) with B-cell non-Hodgkin lymphoma (B-NHL). Of the total cohort, 60 patients (57.7%) were female, while 44 patients (42.3%) were male. The median age was 34 years (IQR: 16–52 years), with the B-NHL patients demonstrating a significantly higher median age compared to the B-ALL patients (P < 0.001). Among the study participants, 34 patients (32.7%) were diagnosed with EMD, while 53 patients (51%) had undergone more than 10 cycles of chemotherapy. Additionally, 67 patients (64.4%) had experienced primary refractory disease or recurrence on multiple occasions. A history of previous allogeneic HCT was observed in 27 patients (26%).

In total, 62 (77.5%) B-ALL patients and 15 (62.5%) B-NHL patients achieved an overall response. 71 patients (68.3%) experienced CRS, with 25 patients (24%) presenting with severe CRS, defined as grade 3–4 CRS. Notably, the incidence of CRS was more prevalent in the B-ALL group compared to the B-NHL group. Table 1 presents comprehensive patient data for each therapeutic group.

**Table 1:** Baseline characteristics of patients on CAR-T cell therapy

Characteristics	Total (n = 104)	B-ALL (n = 80)	B-NHL (n = 24)	P value
Sex, n (%)				
Male	60 (57.7)	42 (52.5)	18 (75.0)	0.050
Female	44 (42.3)	38 (47.5)	6 (25.0)	
Age, years, median (IQR)	34 (16, 52)	24 (14, 49)	52 (38, 58)	** < 0.001**
EMD^a^, n (%)				0.567
No	70 (67.3)	55 (68.8)	15 (62.5)	
Yes	34 (32.7)	25 (31.2)	9 (37.5)	
Chemotherapy^b^, n (%)				0.410
No	51 (49.0)	41 (51.2)	10 (41.7)	
Yes	53 (51.0)	39 (48.8)	14 (58.3)	
Refractory/relapsed^c^, n (%)				0.217
No	37 (35.6)	31 (38.8)	6 (25.0)	
Yes	67 (64.4)	49 (61.3)	18 (75.0)	
Prior HCT^d^, n (%)				0.086
No	77 (74.0)	56 (70.0)	21 (87.5)	
Yes	27 (26.0)	24 (30.0)	3 (12.5)	
Overall response^e^, n (%)	77 (74.0)	62 (77.5)	15 (62.5)	0.142
CRS grading, n (%)				**0.014**
Grade 0	33 (31.7)	20 (25.0)	13 (54.2)	
Grade 1–2	46 (44.2)	37 (46.2)	9 (37.5)	
Grade 3–4	25 (24.0)	23 (28.7)	2 (8.3)	
ICANS, n (%)				1.000
No	99 (95.2)	76 (95.0)	23 (95.8)	
Yes	5 (4.8)	4 (5.0)	1 (4.2)	

*B-ALL* B-cell Acute Lymphoblastic Leukemia, *B-NHL* B-cell Non-Hodgkin Lymphoma, *CRS* cytokine release syndrome, *ICANS* immune effector cell-associated neurotoxicity syndrome

^a^Indicates whether patients had extramedullary diseases

^b^Indicates whether the patient has received chemotherapy more than 10 times

^c^Indicates whether the patient has experienced more than 2 times of relapsed or refractory to treatment

^d^Indicates whether the patient has received allogeneic hematopoietic cell transplantation in the past

^e^Overall response was assessed within two months after CAR-T cell infusion (CTI) in B-ALL and within three months after CTI in B-NHL. Bold values are statistically significant

### Infection incidence within the initial 30 days after CTI

A total of 38 patients (36.5%), comprising 33 with B-ALL and 5 with B-NHL, experienced infection within the initial 30 days post CTI. The specific infection events are detailed in Table 2. Among patients with infections, bacterial microorganisms were the most prevalent (n = 30, 78.9%), while fungal (n = 5, 13.2%) and viral (n = 3, 7.9%) infections were relatively infrequent. The lung was the primary site of infection (n = 17, 44.7%), followed by the bloodstream (n = 11, 28.9%). Other sites (n = 0, 26.3%) included skin and soft tissue (n = 6), intestinal tract (n = 2), and urinary tract (n = 2) infections. A total of 21 cases (55.3%) were clinically diagnosed as non-severe infections, whereas all severe infections (n = 17, 44.7%) were bacterial in nature, resulting in four fatalities.

**Table 2:** Incidence of infections during the first 30 days after CAR-T cell infusion

Type of infection	Patients No.	Total, n = 104, (%)	Infection, n = 38, (%)
Any infection	38	36.5	100
Infection microorganisms			
Bacteria	30	28.8	78.9
Virus	3	2.9	7.9
Fungi	5	4.8	13.2
Infection site			
Lung	17	16.3	44.7
Bloodstream	11	10.6	28.9
Others	10	9.6	26.3
Severity of infection			
Non-severe	21	20.2	55.3
Severe	17	16.3	44.7

The day of diagnostic testing was considered the onset time for each respective infection event, and cumulative incidence curves were employed to illustrate the time points for each incidence of infection. Infections primarily manifested within the initial 2 weeks post CTI (median: day 8; IQR: day 5 to day 10), which was delayed compared to the onset of CRS (median day 1, IQR: day 1 to day 3). Fungal and viral infections were identified on day 8 (median, IQR: 7–20) and day 9 (IQR: 7–24), occurring later than bacterial infections on day 7 (median, IQR: 5–10).

**Fig. 2:**
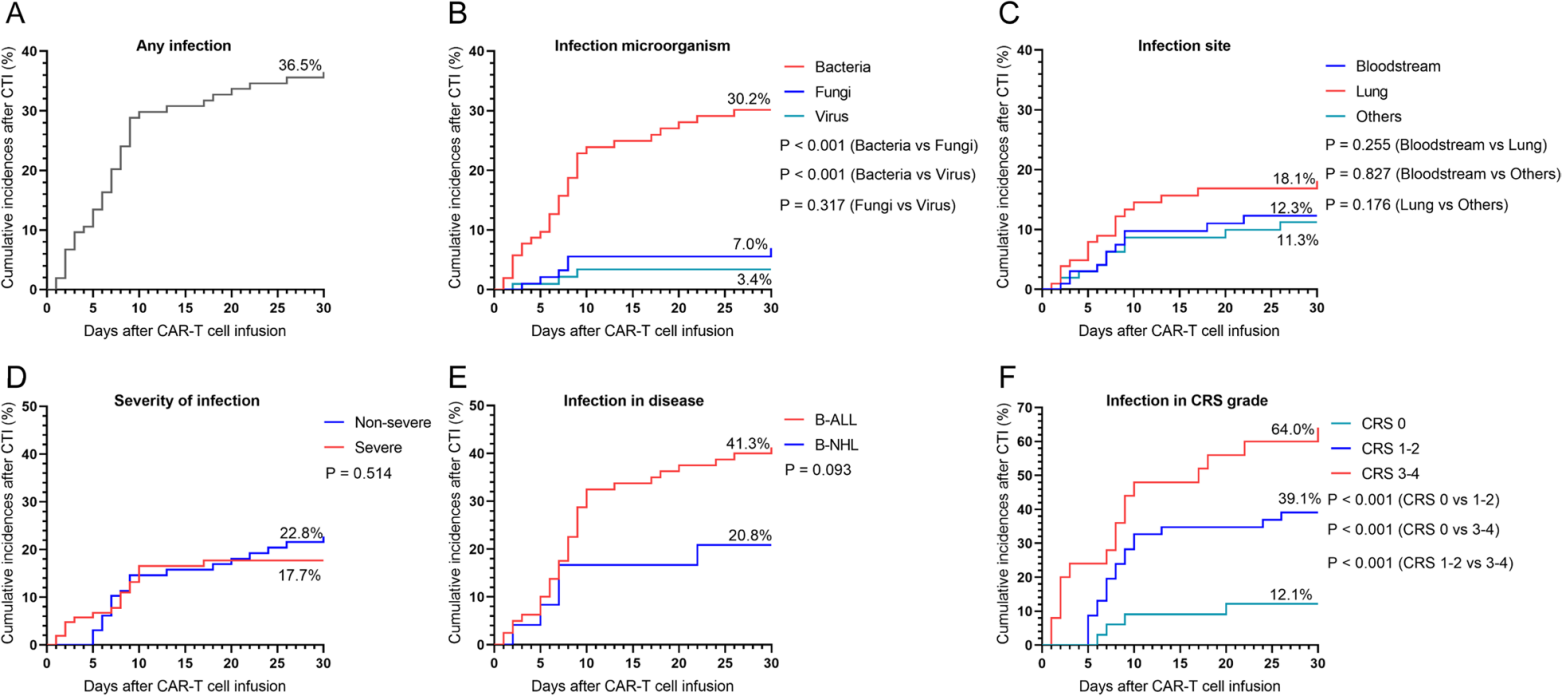
Cumulative-incidence curves of time-to-first infection within 30 days after CAR-T cell infusion(CTI). **A** Cumulative incidences of any infection among all patients (n = 104). **B**–**F** Cumulative incidences of infection in terms of infectious microorganisms, infectious area, severity of infection, infection disease group, and infection in CRS

The cumulative 30-day incidence of infection was 36.5%, with the bacterial incidence higher than that of fungal and viral infections (P < 0.001) (bacterial, 30.2%; fungal, 7%; viral, 3.4%). Moreover, the cumulative 30-day incidence of infection in CRS grade 3–4 was higher than that in CRS grade 1–2 or no CRS (P < 0.001), and CRS grade 1–2 was higher than that of no CRS (P < 0.001) (non-CRS, 12.1%; CRS 1–2, 39.1%; CRS 3–4, 64.0%). No statistically significant differences were observed in terms of infection site, severity of infection, and infection in disease. As shown in Fig. 2A–F.

To further investigate the relationship between patients' clinical characteristics and the occurrence of infection and CRS, we conducted separate comparisons based on infection and CRS grading groups (Tables S1 and S2). The results revealed a higher proportion of patients with prior allogeneic HCT and severe CRS experienced infections. Conversely, patients who responded to treatment exhibited a lower incidence of infections. Moreover, B-ALL patients showed a higher incidence of severe CRS. However, no significant differences were observed in the remaining clinical characteristics regarding the incidence of infection and CRS.

Finally, We evaluated clinical characteristics to determine whether some factors were associated with the risk of infection within 30 days after CTI. Our analysis revealed that prior allogeneic HCT was an independent risk factor for infection (HR: 4.432 [1.262–15.565], P = 0.020). Moreover, CRS was identified as an independent risk factor for both infection (HR: 2.903 [1.577–5.345], P < 0.001) and severe infection (HR: 9.040 [2.256–36.232], P < 0.001) in these patients (refer to Tables S3 and S4 for details).

### Predictive biomarkers for early infection after CTI

**Fig. 3:**
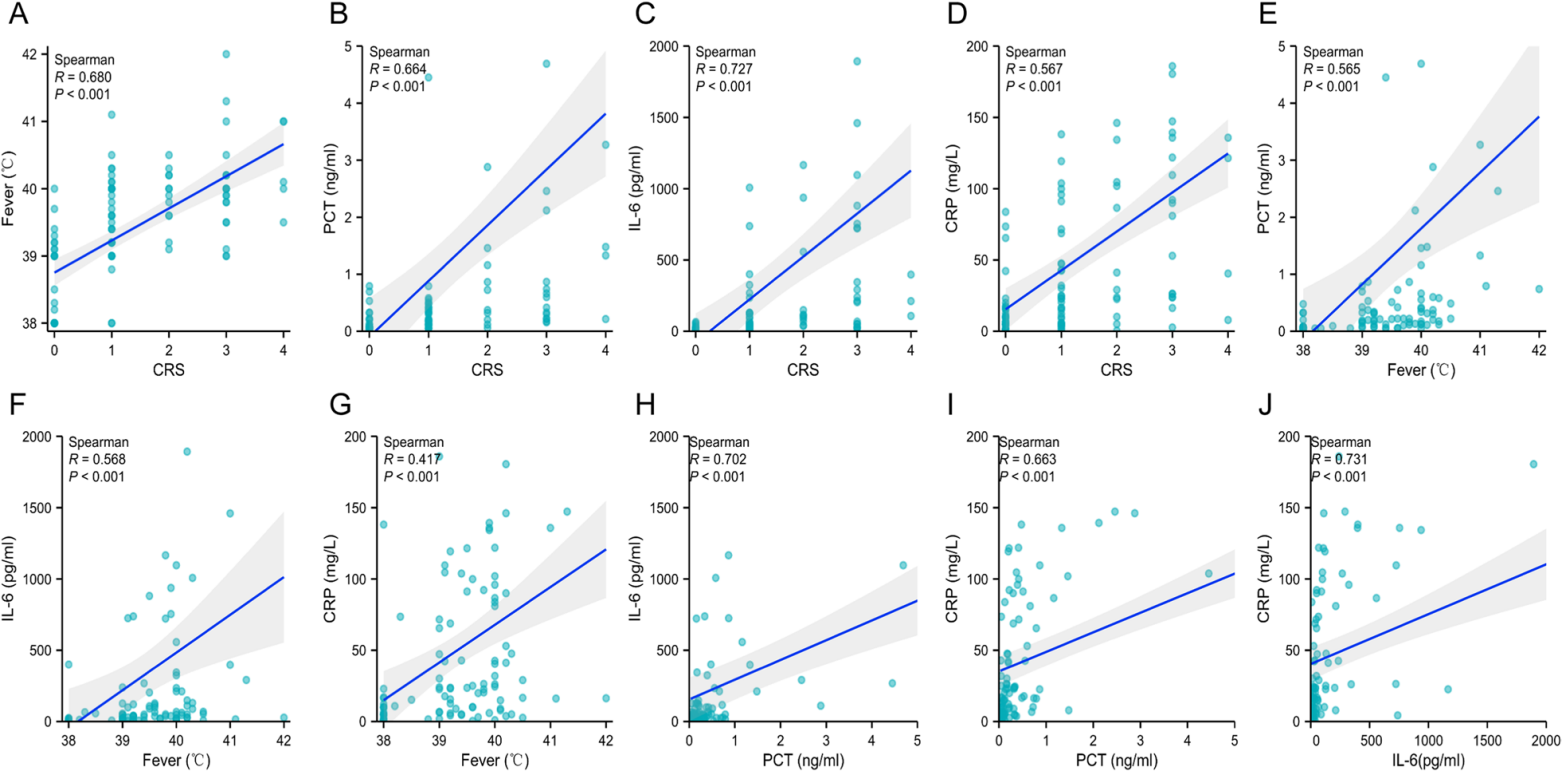
Correlation analysis of clinical indicators. **A**–**J** The correlation among the indicators CRS, fever, PCT, IL-6, and CRP

To assess the efficacy of inflammatory markers for early infection diagnosis in CAR-T therapy patients, we measured serum inflammatory indicators (PCT, IL-6, and CRP) at the onset of fever following CTI. Patients with subsequent infections exhibited significantly elevated levels of fever, PCT, IL-6, and CRP compared to those without infections (P < 0.001, Table 3). We analyzed the correlations among CRS, fever, PCT, IL-6, and CRP (Fig. 3). Our findings revealed a significant positive correlation between CRS and fever, as well as with inflammatory markers. Particularly noteworthy was the strong correlation observed between CRS and IL-6 (R = 0.727, P < 0.001). Furthermore, fever demonstrated positive correlations with all three inflammatory markers.

**Table 3:** Clinical indicators at the onset of fever after CAR-T cell infusion

Characteristics	Total (n = 104)	Non-infection (n = 66)	Infection (n = 38)	P value
Sex, n (%)				0.657
Male	60 (57.7)	37 (56.1)	23 (60.5)	
Female	44 (42.3)	29 (43.9)	15 (39.5)	
Age, years, median (IQR)	34 (16, 52)	35 (15, 53)	32 (16, 51)	0.989
Fever, (℃) median (IQR)	39.5 (39.0, 40.0)	39.2 (38.0, 39.9)	39.9 (39.5, 40.2)	** < 0.001**
PCT, (ng/ml) median (IQR)	0.219 (0.082, 0.669)	0.118 (0.059, 0.310)	0.828 (0.383, 4.630)	** < 0.001**
IL-6, (pm/ml) median (IQR)	46.5 (14.7, 205.0)	26.5 (6.7, 53.0)	222.5 (67.3, 746.9)	** < 0.001**
CRP, (mg/l), median (IQR)	23.0 (6.7, 87.5)	15.1 (5.0, 25.8)	100.9 (27.0, 137.6)	** < 0.001**

*PCT* procalcitonin, *IL-6* interleukin-6, *CRP* C-reactive protein

Bold values are statistically significant

**Fig. 4:**
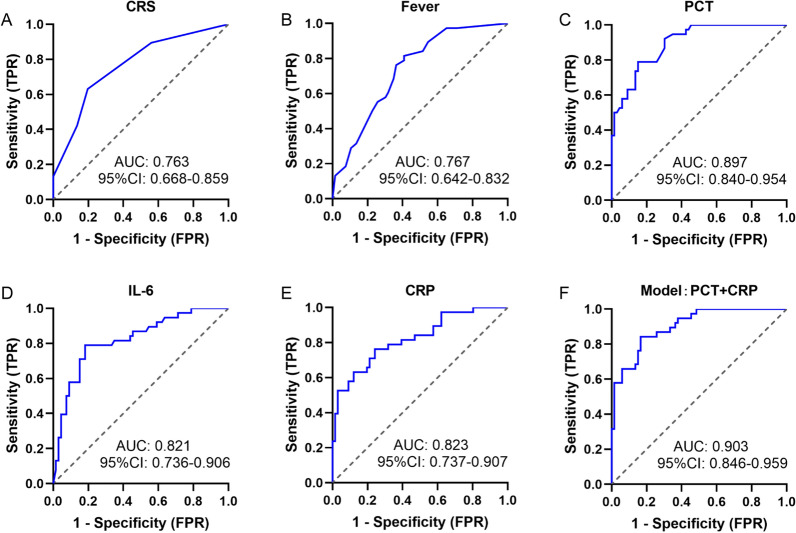
Predictive biomarkers for early infection after CTI. **A**–**E** Receiver operating characteristic (ROC) curve for CRS, fever, PCT, IL-6, CRP in predicting infection. **F** ROC curve for a model based on PCT and CRP in predicting infection

Subsequently, we assessed the diagnostic capabilities of CRS, fever, PCT, IL-6, and CRP in the early diagnosis of infection in febrile patients after CTI using the ROC curve and the Youden’s index. As depicted in Fig. 4A–E, PCT exhibited the highest discriminative value with an AUC of 0.897 (95% confidence interval [CI], 0.840–0.954; P < 0.001), followed by CRP (AUC 0.823 [0.737–0.907]; P < 0.001), and IL-6 (AUC 0.821 [0.736–0.906]; P < 0.001). The performance of each indicator in diagnosing infection is summarized in Table 4. Fever symptoms demonstrated the highest sensitivity (81.6%) with a cut-off value 39.25 ℃. PCT exhibited the best individual biomarker in terms of specificity (84.8%) and accuracy (82.7%) with a cut-off value 0.355 ng/ml.

**Table 4:** Clinical performance of indicators in diagnosing infection after CAR-T cell infusion

Indicators	Cut-off^a^	Sensitivity, %	Specificity, %	Accuracy, %	PPV, %	NPV, %	Youden Index
CRS, (grade)	1.5	63.2	80.3	74.0	64.9	79.1	0.435
Fever, (℃)	39.25	81.6	59.1	67.3	53.4	84.8	0.407
PCT, (ng/ml)	0.355	78.9	84.8	82.7	75.0	87.5	0.638
IL-6, (pm/ml)	65.55	78.9	81.8	80.8	71.4	87.1	0.608
CRP, (mg/l)	26.18	76.3	75.8	76.0	64.4	84.7	0.521
Model^b^	− 1.011	84.2	83.3	83.7	74.4	90.2	0.675

*CRS* cytokine release syndrome, *PCT* Procalcitonin, *IL-6* Interleukin-6, *CRP* C-reactive protein, *PPV* positive predictive value, *NPV* negative predictive value

^a^Cut-off values were determined using the Youden’s index (J = max [sens + spec–1])

^b^A model based on PCT and CRP in predicting infection

To further evaluate the diagnostic value of these indicators for infection, we employed univariate and stepwise multivariate logistic regression analysis, with an entry criterion of P < 0.1, to establish the diagnostic mathematical model. PCT and CRP were included in the model based on the current dataset (Table S5). Furthermore, the model, evaluated using the ROC curve and the Youden’s index, showed an AUC of 0.903 [0.846–0.959], higher than that of any single indicator alone, with a sensitivity of 84.2% and specificity of 83.3% (Fig. 4F, Table 4).

### Predictive biomarkers for early severe infection after CTI

**Fig. 5:**
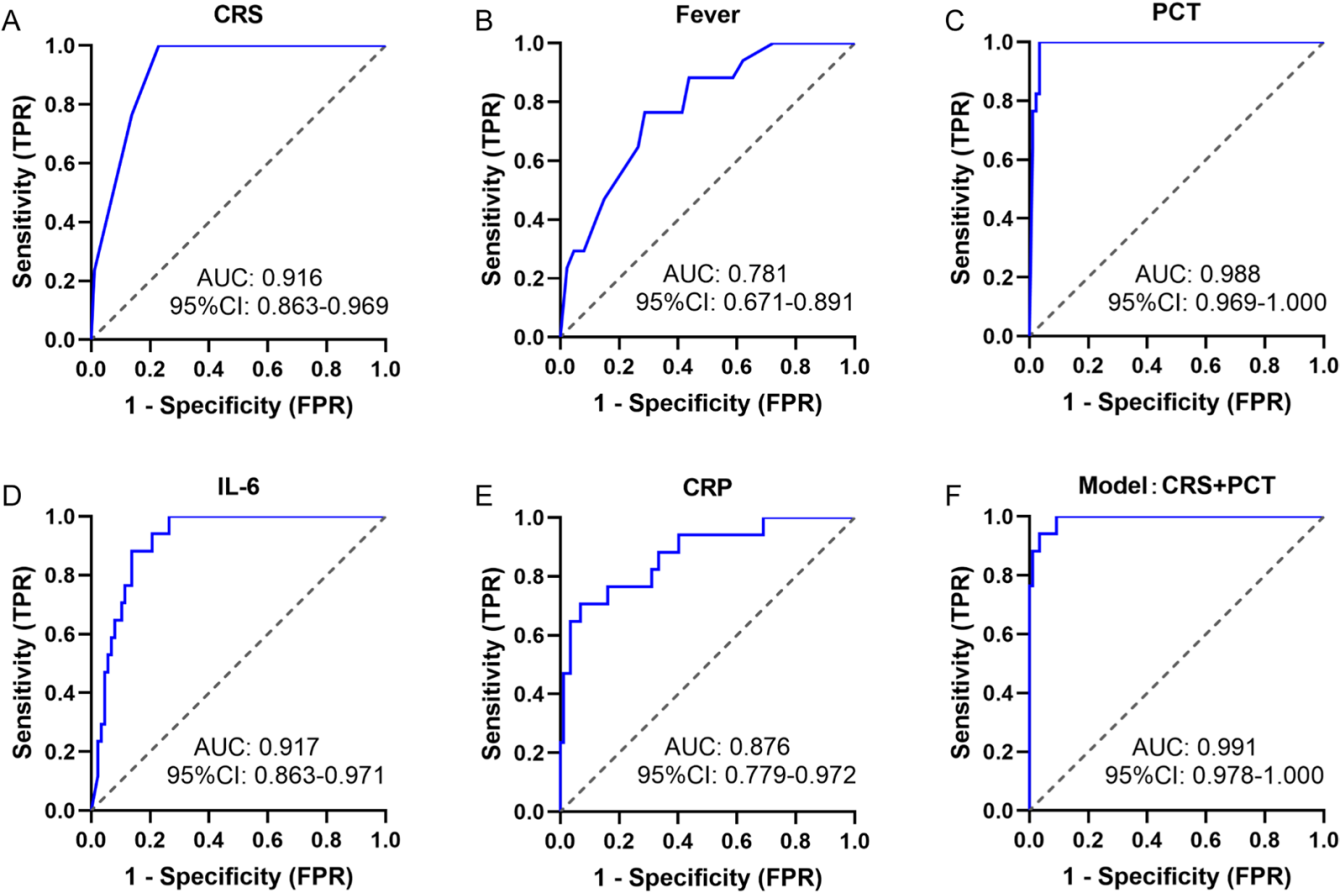
Predictive biomarkers for early severe infection after CTI. **A**–**E** Receiver operating characteristic (ROC) curve for CRS, fever, PCT, IL-6, CRP in predicting severe infection. **F** ROC curve for a model based on CRS and PCT in predicting severe infection

We conducted a comprehensive assessment of the diagnostic potential of CRS, fever, PCT, IL-6, and CRP for the early detection of severe infection in febrile patients following CTI. As illustrated in Fig. 5A–E, PCT exhibited the highest discriminative value with an AUC of 0.988 (0.969–1.000; P < 0.001), followed by IL-6 (AUC 0.917 [0.823–0.971]; P < 0.001), and CRS (AUC 0.916 [0.963–0.969]; P < 0.001). The diagnostic performance of each indicator in identifying severe infection is summarized in Table 5. Notably, the data indicated that PCT exhibited the highest performance as an individual biomarker, demonstrating 100% sensitivity and 96.6% specificity with a cut-off value 0.865 ng/ml.

**Table 5:** Clinical performance of indicators in diagnosing severe infection after CAR-T cell Infusion

Indicators	Cut-off^a^	Sensitivity, %	Specificity, %	Accuracy, %	PPV, %	NPV, %	Youden Index
CRS, (Grade)	1.5	100.0	77.0	80.8	45.9	100.0	0.770
Fever, (℃)	39.85	76.5	71.3	72.1	34.2	93.9	0.477
PCT, (ng/ml)	0.865	100.0	96.6	97.1	85.0	100.0	0.966
IL-6, (pm/ml)	175.5	88.2	86.2	86.5	55.6	97.4	0.744
CRP, (mg/l)	107.1	70.6	93.1	89.4	66.7	94.2	0.637
Model^b^	− 2.68	100.0	90.8	92.3	68.0	100.0	0.908

*CRS* cytokine release syndrome, *PCT* procalcitonin, *IL-6* interleukin-6, *CRP* C-reactive protein, *PPV* positive predictive value, *NPV* negative predictive value

^a^Cut-offs were determined using the Youden’s index (J = max [sens + spec − 1])

^b^A model based on PCT and CRS in predicting severe infection

To further assess the diagnostic value of these indicators for severe infection, we conducted univariate and stepwise multivariate logistic regression analyses, with an entry criterion of P < 0.1, to establish the diagnostic mathematical model. CRS and PCT were identified as key factors included in the model based on the current dataset (Table S6). Furthermore, the model demonstrated an AUC of 0.991 [0.978–1.000], with a sensitivity of 100% and specificity of 90.8% (Fig. 5F, Table 5).

### Predictive biomarkers for early bacterial infection after CTI

**Fig. 6:**
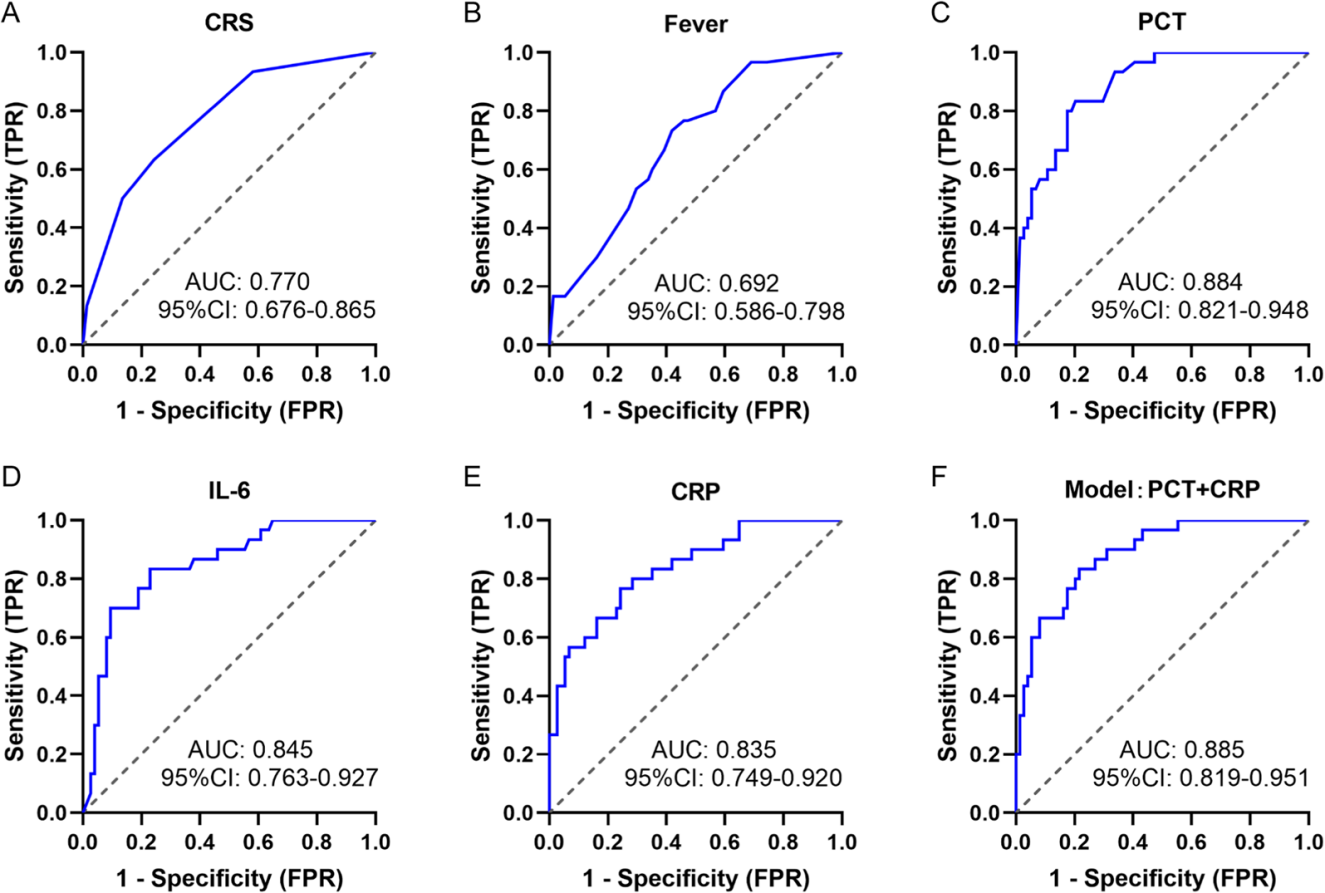
Predictive biomarkers for early bacterial infection after CTI. **A**–**E** Receiver operating characteristic (ROC) curve for CRS, fever, PCT, IL-6, CRP in predicting bacterial infection. **F** ROC curve for a model based on PCT and CRP in predicting bacterial infection

As the predominant infectious complication observed is bacterial infections, it is imperative to assess the diagnostic value of these markers specifically for bacterial infections. As illustrated in Fig. 6A–E, PCT exhibited the highest discriminative value with an AUC of 0.884 (0.821–0.948; P < 0.001), followed by IL-6 (AUC 0.845 [0.763–0.927]; P < 0.001), and CRP (AUC 0.835 [0.749–0.920]; P < 0.001). The diagnostic performance of each indicator in identifying bacterial infection is summarized in Table 6. The data indicated that PCT exhibited the highest performance as an individual biomarker, demonstrating 83.3% sensitivity and 79.7% specificity with a cut-off value 0.355 ng/ml.

To further assess the diagnostic value of these indicators for bacterial infection, we conducted univariate and stepwise multivariate logistic regression analyses, with an entry criterion of P < 0.1, to establish the diagnostic mathematical model. PCT and CRP were identified as key factors included in the model based on the current dataset (Table S7). Furthermore, the model demonstrated an AUC of 0.885 [0.819–0.951], with a sensitivity of 83.3% and specificity of 78.4% (Fig. 6F, Table 6).

**Table 6:** Clinical performance of indicators in diagnosing bacterial infection after CAR-T cell infusion

Indicators	Cut-off ^a^	Sensitivity, %	Specificity, %	Accuracy, %	PPV, %	NPV, %	Youden Index
CRS, (Grade)	1.5	63.3	75.7	72.1	51.4	83.6	0.390
Fever, (℃)	39.45	73.3	58.1	62.5	41.5	84.3	0.314
PCT, (ng/ml)	0.355	83.3	79.7	80.8	62.5	92.2	0.631
IL-6, (pm/ml)	139	70.0	90.5	84.6	75.0	88.2	0.605
CRP, (mg/l)	36.2	76.7	75.7	76.0	56.1	88.9	0.523
Model^b^	− 1.561	83.3	78.4	79.8	61.0	92.1	0.617

*CRS* cytokine release syndrome, *PCT* procalcitonin, *IL-6* interleukin-6, *CRP* C-reactive protein, *PPV* positive predictive value, *NPV* negative predictive value

^a^Cut-offs were determined using the Youden’s index (J = max [sens + spec–1])

^b^A model based on PCT and CRP in predicting bacterial infection

**Fig. 7:**
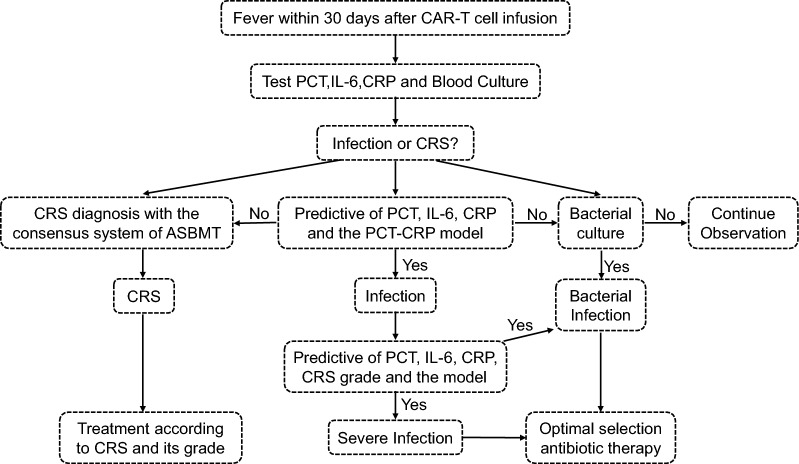
A workflow for rapid identification of early infections in the febrile patients within 30 days after CTI. *CRS* cytokine release syndrome, *PCT* procalcitonin, *IL-6* interleukin-6, *CRP* C-reactive protein, *ASBMT* American Society for Blood and Marrow Transplantation

Based on our analysis, we have developed a workflow for rapid identification of early infections within 30 days of CTI (Fig. 7). When a patient presents with fever within 30 days of CTI, routine evaluations should be conducted to assess the likelihood of CRS and infection. In clinical practice, testing for PCT, IL-6, CRP, and conducting blood cultures are essential for differential diagnosis. The biomarkers, including PCT, IL-6, CRP, and their combination, can help confirm a diagnosis of infection. Prompt initiation of appropriate treatment is crucial for these patients. Additionally, the combination of these biomarkers, alongside CRS grading, can be used to diagnose severe or bacterial infections. Although laboratory results may take some time, a positive bacterial culture provides a definitive diagnosis of bacterial infection and informs the selection of appropriate therapy. If all diagnostic tests yield negative results, patients should continue to be closely monitored.

## Discussion

In this study, we conducted a retrospective analysis of infection characteristics and early detection indicators in febrile patients within 30 days post CTI among 104 patients with R/R B-cell malignancies in our center. Our findings revealed that both prior allogeneic HCT and CRS were identified as independent risk factors for infection in patients after CTI. Moreover, CRS, fever, PCT, IL-6, and CRP emerged as potential indicators for the early detection of infections. Notably, compared to any single indicator alone, a model based on PCT and CRP demonstrated the highest accuracy in predicting infection.

This study provides a comprehensive exploration of the incidence, distribution, severity, and risk factors for infections in febrile patients with B-cell malignancies within the initial 30 days after CTI. Overall, 38 out of 104 patients (36.5%) encountered infections during this period. The incidence and distribution of infections in this cohort closely paralleled observations from other clinical trials involving R/R malignancies within the first month post-CTI [11, 20, 21, 26, 27].

Identifying the clinical and treatment characteristics of patients and understanding their impact on the occurrence of infections after CAR-T therapy may have significant implications for the prevention and management of infections following CAR-T treatment. There have been some reports indicating that immunosuppressive therapy for CRS may heighten the risk of infection [28, 29], a trend consistent with our univariate Cox regression analysis. Previous investigations involving CAR-T cell recipients with B-cell malignancies consistently indicated that severe CRS, graded at level 3 or higher, is associated with elevated infection rates [10, 18, 19]. In our study, we validated these findings, establishing CRS as an independent risk factor for both overall infections and severe infections in this patient cohort. However, the precise mechanism underlying the association between CRS and infection remains elusive. Therefore, further investigations are warranted to elucidate whether elevated levels of proinflammatory and anti-inflammatory cytokines, in conjunction with immunosuppressive therapies, contribute to the elevated risk of infections among patients with CAR-T treated experiencing severe CRS.

However, there is controversy regarding the impact of prior HCT on the occurrence of infections after CAR-T treatment. Joshua A.et al. reported no statistically significant association between prior HCT (including autologous and allogeneic HCT) and infections in patients after CTI [11]. Similarly, two other small-sample-size studies involving 53 and 41 B-cell malignancies patients supported this finding [30, 31]. In contrast, Vora B.et al. identified prior allogeneic HCT as a risk factor for infections after CAR-T therapy [32]. The disparities in research scale and transplant types may explain these conflicting results. Our analysis, conducted with a larger sample size than Surabhi et al., contributes to this discourse by revealing that prior allogeneic HCT independently poses a risk for infection.

Our study contributes to understanding the types and timing of infections, identifying CRS and prior allogeneic HCT as risk factors within the initial 30 days post-CTI. These findings may inform prophylactic and monitoring strategies aimed at preventing and managing infections. This holds substantial significance for improving the efficacy of CAR-T therapy.

In the clinical setting, the resemblance between infection and CAR-T therapy-induced CRS, characterized by heightened inflammatory factors and fever, poses a diagnostic challenge [19, 21, 33]. To address this, we investigated the correlation and diagnostic value of inflammatory markers in the early detection of infection during CAR-T therapy. We systematically measured serum inflammatory indicators, PCT, IL-6, and CRP, at the onset of fever after CTI. Our analysis involves the correlation study of these clinical indicators. Our data revealed a significant correlation between CRS and fever symptoms, along with concurrent alterations in inflammatory factor levels. Likewise, it has been observed that, despite the elevated levels of IL-6 in severe infections, this profile significantly differs from the inflammatory signatures associated with CAR -T cell-induced CRS [21, 34–36]. Similarly, despite infection, CRP levels are also associated with CRS [37]. While PCT stands out as a crucial biomarker for infections, especially severe infections [38, 39]. The results imply the complexity of utilizing inflammatory markers for infection diagnosis, emphasizing the need for further scrutiny and consideration within clinical practice.

Our investigation suggests that despite potential interference from factors like CRS, biomarkers such as PCT, IL-6, and CRP hold promise for early infection detection, particularly in severe cases. Notably, PCT stands out as a pivotal biomarker, particularly valuable in the early identification of both infected patients and severe infection cases. PCT serves as an established sepsis marker and is readily available at most academic centers [38, 40, 41]. Importantly, the utility of PCT as a biomarker to identify early infections in the setting of CRS in our study proved to be valuable.

Moreover, the combined use of PCT and CRP significantly enhances the sensitivity for detecting infections in febrile patients. Simultaneously, the integration of CRS and PCT can be leveraged for the early diagnosis of severe infections. In certain instances, either CRS or infection can be easily managed. However, in other cases, cytokine storms characterized by markedly elevated inflammatory factors can become life-threatening. In such scenarios, a method to differentiate between CRS and infection becomes pivotal. Our study on these biomarkers holds significant clinical potential in addressing this critical differentiation. In forthcoming research, we may explore the development of a clinical algorithm to accurately assess the probability of CRS versus infection, leveraging the insights derived from our findings.

Due to the retrospective analysis and the limited cohort size of our study, further investigations are necessary to identify risk factors for infection post-CTI. Additionally, larger studies with a substantial number of participants and a variety of CAR-T cell types are warranted.

In conclusion, CRS and prior allogeneic HCT were identified as independent risk factors for infection in febrile patients with B-cell malignancies after CTI. Additionally, CRS, fever, PCT, IL-6, and CRP can serve as diagnostic indicators for infection in patients with fever. PCT emerges as a crucial biomarker for the early diagnosis of infected patients and those with severe infections. Significantly, we first reported and established a model that combines PCT and CRP with higher accuracy, offering a novel approach to early infection diagnosis. Our study contributes valuable insights to guide therapeutic decisions for post-CTI patients, aiming to minimize the detrimental effects of infections and optimize the efficacy of CAR-T cell therapy.

### Supplementary Information

Additional file 1: Supplement Table S1. Risk factors for infection within the first 30 days after CAR-T cell infusion. Supplement Table S2. Risk factors for severe infection within the first 30 days after CAR-T cell infusion. Supplement Table S3. Univariate and multivariate analysis of indicators with infection after CAR-T cell infusion. Supplement Table S4. Univariate and multivariate analysis of indicators with severe infection after CAR-T cell infusion.

## Data Availability

The datasets used and/or analyzed during the current study are available from the corresponding author on reasonable request. The data included herein are reported within the published article and its supplementary information, and are available from the corresponding author on request.

## Abbreviations

AUC: Area under the ROC curve
B-ALL: B-cell acute lymphoblastic leukemia
B-NHL: B-cell non-Hodgkin lymphoma
CAR-T: Chimeric antigen receptor-modified T
CRP: C-reactive protein
CRS: Cytokine release syndrome
CTCAE: Common terminology criteria of adverse events
CTI: CAR-T cell infusion
CI: Confidence interval
EMD: Extramedullary diseases
HCT: Hematopoietic stem cell transplantation
ICANS: Immune effector cell-associated hematotoxicity
IL-6: Interleukin-6
IQR: Interquartile ranges
PCT: Procalcitonin
ROC: Receiver operating characteristic
R/R: Refractory/relapsed
