# Supplementary material for: Rapid identification of early infections in febrile patients after CD19 target CAR-T cell therapy for B-cell malignancies
Source: J Transl Med. 2024 Jul 2;22:613. doi: 10.1186/s12967-024-05308-2 (PMC11221099; doi:10.1186/s12967-024-05308-2)
Supplement: Supplementary file 1 — Additional file 1: Supplement Table S1. Risk factors for infection within the first 30 days after CAR-T cell infusion. Supplement Table S2. Risk factors for severe infection within the first 30 days after CAR-T cell infusion. Supplement Table S3. Univariate and multivariate analysis of indicators with infection after CAR-T cell infusion. Supplement Table S4. Univariate and multivariate analysis of indicators with severe infection after CAR-T cell infusion. [file 12967_2024_5308_MOESM1_ESM.docx]

Supplement table1. Baseline characteristics of patients receiving CAR-T cell therapy stratified by infection status.

| Characteristics | Total (n = 104) | Non-infection (n = 66) | Infection (n = 38) | P value |
| --- | --- | --- | --- | --- |
| Diagnose, n (%) |  |  |  |  |
| B-ALL | 80 (76.9) | 47 (58.8) | 33 (41.2) | 0.068 |
| B-NHL | 24 (23.1) | 19 (79.2) | 5 (20.8) |  |
| Sex, n (%) |  |  |  | 0.657 |
| Male | 60 (57.7) | 37 (61.7) | 23 (38.3) |  |
| Female | 44 (42.3) | 29 (65.9) | 15 (34.1) |  |
| Age, years, median (IQR) | 34 (16, 52) | 35 (15, 53) | 32 (16, 51) | 0.989 |
| EMD ^a^, n (%) |  |  |  | 0.494 |
| Yes | 34 (32.7) | 20 (58.8) | 14 (41.2) |  |
| No | 70 (67.3) | 46 (65.7) | 24 (34.3) |  |
| Chemotherapy ^b^, n (%) |  |  |  | 0.796 |
| Yes | 53 (51.0) | 33 (62.3) | 20 (37.7) |  |
| No | 51 (49.0) | 33 (64.7) | 18 (35.3) |  |
| Refractory/relapsed ^c^, n (%) |  |  |  | 0.134 |
| Yes | 67 (64.4) | 39 (58.2) | 28 (41.8) |  |
| No | 37 (35.6) | 27 (73.0) | 10 (27.0) |  |
| Prior HCT ^d^, n (%) |  |  |  | **<0.001** |
| Yes | 27 (26.0) | 9 (33.3) | 18 (66.7) |  |
| No | 77 (74.0) | 57 (74.0) | 20 (26.0) |  |
| CRS Grading, n (%) |  |  |  | **<0.001** |
| Grade 0 | 46 (44.2) | 28 (60.9) | 18 (39.1) |  |
| Grade 1-2 | 33 (31.7) | 29 (87.9) | 4 (12.1) |  |
| Grade 3-4 | 25 (24.0) | 9 (36.0) | 16 (64.0) |  |
| ICANS, n (%) |  |  |  | 0.522 |
| Yes | 5 (4.8) | 2 (40.0) | 3 (60.0) |  |
| No | 99 (95.2) | 64 (64.6) | 35 (35.4) |  |
| Efficacy ^e^, n (%) |  |  |  | **< 0.001** |
| CR | 77 (74.0) | 56 (72.7) | 21 (27.3) |  |
| NR | 27 (26.0) | 10 (37.0) | 17 (63.0) |  |

B-ALL, B-cell Acute Lymphoblastic Leukemia; B-NHL, B-cell Non-Hodgkin Lymphoma; CRS, cytokine release syndrome; ICANS, immune effector cell-associated neurotoxicity syndrome. a, indicates whether patients had extramedullary diseases; b, indicates whether the patient has received chemotherapy more than 10 times; c, indicates whether the patient has experienced more than 2 times of relapsed or refractory to treatment; d, indicates whether the patient has received allogeneic hematopoietic cell transplantation in the past; e, overall response was assessed within two months after CAR-T cell infusion (CTI) in B-ALL and within three months after CTI in B-NHL. Bold values are statistically significant.

Supplement table 2. Baseline characteristics of patients receiving CAR-T cell therapy stratified by CRS grading.

| Characteristics | Total  (n = 104) | CRS Grade 0  (n = 33) | CRS Grade 1-2  (n =46) | CRS Grade 3-4  (n = 25) | P value |
| --- | --- | --- | --- | --- | --- |
| Diagnose, n (%) |  |  |  |  | **0.014** |
| B-ALL | 80 (76.9) | 20 (25.0) | 37 (46.2) | 23 (28.7) |  |
| B-NHL | 24 (23.1) | 13 (54.2) | 9 (37.5) | 2 (8.3) |  |
| Sex, n (%) |  |  |  |  | 0.132 |
| Male | 60 (57.7) | 20 (33.3) | 22 (36.7) | 18 (30) |  |
| Female | 44 (42.3) | 13 (29.5) | 24 (54.5) | 7 (15.9) |  |
| Age, years, median (IQR) | 34 (16, 52) | 38 (24, 56) | 31 (11, 55) | 29 (16, 42) | 0.232 |
| EMD ^a^, n (%) |  |  |  |  | 0.423 |
| Yes | 34 (32.7) | 12 (35.3) | 12 (35.3) | 10 (29.4) |  |
| No | 70 (67.3) | 21 (30.0) | 34 (48.6) | 15 (21.4) |  |
| Chemotherapy ^b^, n (%) |  |  |  |  | 0.405 |
| Yes | 53 (51.0) | 14 (26.4) | 24 (45.3) | 15 (28.3) |  |
| No | 51 (49.0) | 19 (37.3) | 22 (43.1) | 10 (19.6) |  |
| Refractory/relapsed ^c^, n (%) |  |  |  |  | 0.289 |
| Yes | 67 (64.4) | 18 (26.9) | 33 (49.3) | 16 (23.9) |  |
| No | 37 (35.6) | 15 (40.5) | 13 (35.1) | 9 (24.3) |  |
| Prior HCT ^d^, n (%) |  |  |  |  | 0.893 |
| Yes | 27 (26.0) | 8 (29.6) | 13 (48.1) | 6 (22.2) |  |
| No | 77 (74.0) | 25 (32.5) | 33 (42.9) | 19 (24.7) |  |
| ICANS, n (%) |  |  |  |  | 0.668 |
| Yes | 5 (4.8) | 1 (20.0) | 2 (40.0) | 2 (40.0) |  |
| No | 99 (95.2) | 32 (32.3) | 44 (44.4) | 23 (23.2) |  |
| Infection |  |  |  |  | **< 0.001** |
| Yes | 38 (36.5) | 4 (10.5) | 18 (47.4) | 16 (42.1) |  |
| No | 66 (63.5) | 29 (43.9) | 28 (42.4) | 9 (13.6) |  |
| Efficacy ^e^, n (%) |  |  |  |  | 0.185 |
| CR | 77 (74.0) | 26 (33.8) | 36 (46.8) | 15 (19.5) |  |
| NR | 27 (26.0) | 7 (25.9) | 10 (37.0) | 10 (37.0) |  |

B-ALL, B-cell Acute Lymphoblastic Leukemia; B-NHL, B-cell Non-Hodgkin Lymphoma CRS, cytokine release syndrome; ICANS, immune effector cell-associated neurotoxicity syndrome. a, indicates whether patients had extramedullary diseases; b, indicates whether the patient has received chemotherapy more than 10 times; c, indicates whether the patient has experienced more than 2 times of relapsed or refractory to treatment; d, indicates whether the patient has received allogeneic hematopoietic cell transplantation in the past; e, overall response was assessed within two months after CAR-T cell infusion (CTI) in B-ALL and within three months after CTI in B-NHL. Bold values are statistically significant

Supplement table 3. Risk factors for infection within the first 30 days after CAR-T cell infusion

| Characteristics | Univariate analysis | |  | Multivariate analysis | |
| --- | --- | --- | --- | --- | --- |
|  | Hazard ratio (95% CI) | P value |  | Hazard ratio (95% CI) | P value |
| Male/Female | 0.907 (0.473 - 1.738) | 0.768 |  | 1.267 (0.580 – 2.679) | 0.553 |
| Age | 1.000 (0.984 - 1.015) | 0.983 |  | 0.991 (0.970 - 1.012) | 0.403 |
| B-ALL/B-NHL | 0.457 (0.178 - 1.172) | 0.103 |  | 1.026 (0.354 - 2.975) | 0.962 |
| EMD ^a^ | 0.850 (0.440 - 1.644) | 0.629 |  | 0.881 (0.421 – 1.844) | 0.737 |
| Chemotherapy ^b^ | 0.904 (0.478 - 1.710) | 0.757 |  | 0.673 (0.314 – 1.441) | 0.308 |
| Refractory/relapsed ^c^ | 0.590 (0.286 - 1.214) | 0.152 |  | 1.054 (0.469 – 2.37) | 0.899 |
| Prior HCT ^d^ | 3.579 (1.269 – 10.094) | **0.016** |  | 4.432 (1.262 – 15.565) | **0.020** |
| CRS | 1.847 (1.433 - 2.380) | **< 0.001** |  | 2.903 (1.577 – 5.345) | **< 0.001** |
| ICANS | 1.933 (0.593 - 6.302) | 0.275 |  | 1.247 (0.338 - 4.606) | 0.740 |
| Tocilizumab/corticosteroids | 3.046 (1.597 - 5.809) | **< 0.001** |  | 3.747 (0.864 – 16.252) | 0.078 |

B-ALL, B-cell Acute Lymphoblastic Leukemia; B-NHL, B-cell Non-Hodgkin Lymphoma; CRS, cytokine release syndrome; ICANS, immune effector cell-associated neurotoxicity syndrome. a, indicates whether patients had extramedullary diseases; b, indicates whether the patient has received chemotherapy more than 10 times; c, indicates whether the patient has experienced more than 2 times of relapsed or refractory to treatment; d, indicates whether the patient has received allogeneic hematopoietic cell transplantation in the past. Bold values are statistically significant.

Supplement table 4. Risk factors for severe infection within the first 30 days after CAR-T cell infusion

| Characteristics | Univariate analysis | |  | Multivariate analysis | |
| --- | --- | --- | --- | --- | --- |
|  | Hazard ratio (95% CI) | P value |  | Hazard ratio (95% CI) | P value |
| Male/Female | 1.231 (0.475 - 3.191) | 0.669 |  | 3.510 (0.883 - 13.958) | 0.075 |
| Age | 0.988 (0.965 - 1.012) | 0.344 |  | 0.983 (0.944 - 1.023) | 0.387 |
| B-ALL/B-NHL | 0.195 (0.026 - 1.472) | 0.113 |  | 0.961 (0.111 - 8.311) | 0.971 |
| EMD ^a^ | 0.699 (0.266 - 1.837) | 0.468 |  | 2.269 (0.572 - 9.004) | 0.244 |
| Chemotherapy ^b^ | 0.705 (0.268 - 1.853) | 0.478 |  | 0.797 (0.210 - 3.034) | 0.740 |
| Refractory/relapsed ^c^ | 0.526 (0.171 - 1.612) | 0.261 |  | 1.206 (0.291 - 5.001) | 0.796 |
| Prior HCT ^d^ | 0.595 (0.171 - 2.069) | 0.414 |  | 1.473 (0.203 - 10.693) | 0.702 |
| CRS | 4.105 (2.401 - 7.018) | **< 0.001** |  | 9.040 (2.256 - 36.232) | **< 0.001** |
| ICANS | 1.283 (0.170 - 9.676) | 0.809 |  | 0.297 (0.029 - 3.078) | 0.309 |
| Tocilizumab/corticosteroids | 14.290 (4.642 - 43.984) | **< 0.001** |  | 3.569 (0.273 - 46.712) | 0.332 |

B-ALL, B-cell Acute Lymphoblastic Leukemia; B-NHL, B-cell Non-Hodgkin Lymphoma; CRS, cytokine release syndrome; ICANS, immune effector cell-associated neurotoxicity syndrome. a, indicates whether patients had extramedullary diseases; b, indicates whether the patient has received chemotherapy more than 10 times; c, indicates whether the patient has experienced more than 2 times of relapsed or refractory to treatment; d, indicates whether the patient has received allogeneic hematopoietic cell transplantation in the past. Bold values are statistically significant.

Supplement table 5. Univariate and multivariate analysis of indicators with infection after CAR-T cell infusion

| Characteristics | Univariate analysis | |  | Multivariate analysis | |
| --- | --- | --- | --- | --- | --- |
|  | Odds Ratio (95% CI) | P value |  | Odds Ratio (95% CI) | P value |
| Male/Female | 0.832 (0.369 - 1.874) | 0.657 |  |  |  |
| Age | 1.000 (0.980 - 1.020) | 0.978 |  |  |  |
| B-ALL/B-NHL | 0.375 (0.127 - 1.105) | 0.075 |  | 1.105 (0.261 - 4.684) | 0.892 |
| CRS | 2.352 (1.596 - 3.465) | **< 0.001** |  | 0.854 (0.460 - 1.585) | 0.616 |
| Fever | 3.415 (1.835 - 6.356) | **< 0.001** |  | 1.940 (0.790 - 4.769) | 0.148 |
| PCT | 15.449 (3.315 - 72.000) | **< 0.001** |  | 10.926 (1.461 - 81.731) | **0.020** |
| IL-6 | 1.002 (1.001 - 1.004) | **0.002** |  | 0.999 (0.997 - 1.001) | 0.229 |
| CRP | 1.026 (1.015 - 1.037) | **< 0.001** |  | 1.020 (1.005 - 1.035) | **0.007** |

B-ALL, B-cell Acute Lymphoblastic Leukemia; B-NHL, B-cell Non-Hodgkin Lymphoma; CRS, cytokine release syndrome; PCT, Procalcitonin; IL-6, Interleukin-6; CRP, C-reactive protein. Bold values are statistically significant.

Supplement table 6. Univariate and multivariate analysis of indicators with severe infection after CAR-T cell infusion

| Characteristics | Univariate analysis | |  | Multivariate analysis | |
| --- | --- | --- | --- | --- | --- |
|  | Odds Ratio (95% CI) | P value |  | Odds Ratio (95% CI) | P value |
| Male/Female | 1.259 (0.444 - 3.575) | 0.665 |  |  |  |
| Age | 0.988 (0.962 - 1.014) | 0.359 |  |  |  |
| B-ALL/B-NHL | 0.174 (0.022 - 1.386) | 0.099 |  | 0.292 (0.000 - 188.570) | 0.710 |
| CRS | 5.704 (2.599 - 12.520) | **< 0.001** |  | 7.435 (1.112 - 49.713) | **0.039** |
| Fever | 5.051 (1.990 - 12.822) | **< 0.001** |  | 0.986 (0.076 - 12.832) | 0.991 |
| PCT | 13.556 (4.604 - 39.910) | **< 0.001** |  | 13.330 (2.532 - 70.193) | **0.002** |
| IL-6 | 1.002 (1.001 - 1.003) | **< 0.001** |  | 0.999 (0.996 - 1.001) | 0.342 |
| CRP | 1.026 (1.015 - 1.037) | **< 0.001** |  | 1.008 (0.977 - 1.039) | 0.629 |

B-ALL, B-cell Acute Lymphoblastic Leukemia; B-NHL, B-cell Non-Hodgkin Lymphoma; CRS, cytokine release syndrome; PCT, Procalcitonin; IL-6, Interleukin-6; CRP, C-reactive protein. Bold values are statistically significant.

Supplement table 7. Univariate and multivariate analysis of indicators with bacterial infection after CAR-T cell infusion

| Characteristics | Univariate analysis | |  | Multivariate analysis | |
| --- | --- | --- | --- | --- | --- |
|  | Odds Ratio (95% CI) | P value |  | Odds Ratio (95% CI) | P value |
| Male/Female | 0.719 (0.301 - 1.721) | 0.459 |  |  |  |
| Age | 0.998 (0.977 - 1.020) | 0.888 |  |  |  |
| B-ALL/B-NHL | 0.280 (0.077 - 1.024) | 0.054 |  | 0.558 (0.110 - 2.819) | 0.480 |
| CRS | 2.345 (1.575 - 3.490) | **< 0.001** |  | 1.186 (0.662 - 2.125) | 0.567 |
| Fever | 2.752 (1.491 - 5.079) | **0.001** |  | 1.217 (0.501 - 2.961) | 0.664 |
| PCT | 4.098 (2.182 - 7.696) | **< 0.001** |  | 2.169 (0.968 - 4.860) | 0.060 |
| IL-6 | 1.002 (1.001 - 1.003) | **< 0.001** |  | 1.000 (0.998 - 1.001) | 0.635 |
| CRP | 1.024 (1.014 - 1.034) | **< 0.001** |  | 1.017 (1.004 - 1.029) | **0.008** |

B-ALL, B-cell Acute Lymphoblastic Leukemia; B-NHL, B-cell Non-Hodgkin Lymphoma; CRS, cytokine release syndrome; PCT, Procalcitonin; IL-6, Interleukin-6; CRP, C-reactive protein. Bold values are statistically significant.
